# Clinical Characteristics of Spinal Levobupivacaine: Hyperbaric Compared with Isobaric Solution

**DOI:** 10.1100/2012/169076

**Published:** 2012-04-24

**Authors:** Vimolluck Sanansilp, Travuth Trivate, Phakaporn Chompubai, Shusee Visalyaputra, Pattipa Suksopee, Ladda Permpolprasert, Benno von Bormann

**Affiliations:** Department of Anesthesiology, Faculty of Medicine Siriraj Hospital, Mahidol University, Bangkok 10700, Thailand

## Abstract

We performed a prospective, double-blinded study in 20 patients undergoing gynecologic surgery with lower abdominal incision, to investigate characteristics of intrathecal hyperbaric levobupivacaine compared with isobaric levobupivacaine. We randomly assigned them to receive 3 mL of either isobaric or hyperbaric 0.42% levobupivacaine intrathecally. We found that hyperbaric levobupivacaine, compared with isobaric levobupivacaine, spread faster to T10 level (2.8 ± 1.1 versus 6.6 ± 4.7 minutes, *P* = 0.039), reached higher sensory block levels at 5 and 15 minutes after injection (T8 versus L1, *P* = 0.011, and T4 versus T7, *P* = 0.027, resp.), and had a higher peak level (T4 versus T8, *P* = 0.040). Isobaric levobupivacaine caused a wider range of peak levels (L1 to C8) compared with hyperbaric form (T7 to T2). The level of T4 or higher reached 90% in the hyperbaric group compared with 20% in the isobaric group (*P* = 0.005). Our results suggest that hyperbaric levobupivacaine was more predictable for sensory block level and more effective for surgical procedures with lower abdominal approach. Hyperbaric levobupivacaine seems to be suitable, but the optimal dosage needs further investigation.

## 1. Introduction

An increasing number of studies compare spinal levobupivacaine, an S(−)-enantiomer of bupivacaine, with racemic bupivacaine or other local anesthetics, either isobaric or hyperbaric, for obstetrics, orthopedics, herniorrhaphy, and transurethral surgeries [1–4]. Only one study [5] compared the isobaric to the hyperbaric form of the same agent. However there are no conclusive data yet, whether one form is superior to the other, especially in lower abdominal surgery. Up to now there is no study about spinal anesthesia in gynecological surgery with abdominal incision which requires a higher level of sensory block. Therefore the quality of anesthesia, sensory and motor block characteristics and hemodynamics in patients requiring a higher level of spinal block for lower abdominal approach after either hyperbaric or isobaric levobupivacaine are of particular interest. In addition, anesthesiologists in our country generally use the hyperbaric form of local anesthetics for intra-abdominal surgery but the manufactured hyperbaric form of levobupivacaine is not available; so it is interesting to know whether it is worth making it hyperbaric.

The objectives of this study are to investigate specific blocking characteristics, surgical quality, and side effects of intrathecal hyperbaric levobupivacaine compared with isobaric levobupivacaine for gynecologic surgery with abdominal incision.

## 2. Methods

This prospective randomized, double-blinded study was approved by Siriraj Institutional Review Board (Si211/2006) and registered with ClinicalTrials.gov (NCT01349751). Written informed consent was obtained from 20 ASA I–III patients, aged 18–70 years, who were scheduled for elective gynecologic surgery including total abdominal hysterectomy with or without uni/bilateral salpingo-oophorectomy, uni/bilateral ovarian cystectomy, or myomectomy, at Siriraj Hospital gynecologic operating rooms. Patients with contraindications for spinal block, BMI more than 35 kg/m^2^, and height less than 150 cm were excluded.

Under standard monitoring all patients received 15 mL/kg of lactated Ringer's solution intravenously during the insertion of epidural catheter and completed before inducing spinal block. Epidural catheter 16G was inserted at the L2-3 interspace in the right lateral decubitus position, midline approach and retained for postoperative epidural opioid analgesia and as a rescue treatment in case of inadequate spinal anesthesia. Preoperatively no epidural anesthetics were administered.

Using a computer-generated randomization sequence with sealed envelopes, patients were allocated to receive either isobaric or hyperbaric levobupivacaine (Chirocaine, Abbott Laboratories, Nycomed Pharma AS, Norway). Both solutions were aseptically prepared immediately before injection by an anesthesiologist, who was not involved in further patient care, by adding either 0.48 mL of 50% glucose (240 mg to make 8% glucose) or saline solution to 2.52 mL of 0.5% isobaric levobupivacaine (12.6 mg), the only preparation of levobupivacaine we have, to achieve a final concentration of 0.42% levobupivacaine in 3 mL.

After inserting an epidural catheter, we performed spinal anesthesia to the patient in the same position on the horizontal plane operating table, at L3-4 interspace, midline approach, using a 26G Quincke spinal needle (Becton Dickinson, Madrid, Spain). The anesthetic was injected with approximately 0.1 mL/sec. Cerebrospinal fluid (CSF) was aspirated once at the end of the injection to confirm the position of the needle. The time finishing the injection was considered “time zero.” The patient was immediately turned supine.

An investigator blinded to the type of solution recorded the quality of sensory block, checking pinprick sensation every single minute for 20 minutes, then at 30, 45, and 60 minutes after injection, and then every 30 minutes until sensory regression to T10 level. The peak levels of sensory block, time to T4 level, time-to-peak sensory block, 2-segment regression time, and regression time to T10 level were determined.

Motor blockade, onset as well as regression, was evaluated concurrently with sensory blockade, using a modified Bromage score 0–3 (0, no motor block; 1, unable to raise extended legs, able to move knees and feet; 2, unable to raise extended legs and move knees, able to move feet; 3, complete motor block of the lower limbs). We defined adequate motor block (score ≥2) and/or no response to pain as tested by the surgeon as readiness for surgical incision.

Basic hemodynamic variables were recorded frequently. Hypotension was defined as a decrease in systolic blood pressure by ≥20% from baseline value or to <100 mmHg and was treated with a rapid IV infusion and an incremental IV bolus of either ephedrine (6–12 mg) if heart rate was <100 bpm or norepinephrine (4 mcg) if heart rate was ≥100 bpm. Bradycardia was defined as heart rate <45 bpm and treated with IV atropine 0.01 mg/kg.

Fifteen minutes after spinal injection 20–30 mg pethidine and/or 1-2 mg midazolam could be given intravenously, if required. If a patient felt uncomfortable with some recurrent pain, ketamine 10–20 mg IV could be given incrementally, additionally. Thirty minutes before the estimated end of operation, morphine 3 or 4 mg (for patients >60 or ≤60 years old, resp.) in normal saline solution 4 mL was given via epidural catheter. All medications and time given were recorded.

Surgeons were asked to rate the convenience of surgical condition. Spinal block was considered successful when surgery was completed without converting to epidural or general anesthesia. Patients assessed the quality of anesthesia using a grading system 0–3, sedated (0 (worst), discomfort because of pain; 1 (poor), discomfort because of feeling intense pressure or traction; 2 (fair), comfortable but experienced pressure and traction; 3 (good), comfortable without any feeling, and sedated, no grading possible). This was done 4 times whenever possible, that is, at skin incision time, 1 hour and 2 hours after injection of levobupivacaine, and at skin suture time.

We determined a T4 sensory block level within 10–15 minutes to estimate sample size, as T4 is adequate for intra-abdominal surgery with peritoneal traction, and 15 minutes is an interval suitable for clinical routine. After investigating ten (five in each group) randomized patients as a pilot group, time to T4 level in the hyperbaric group had the mean ± SD of 9.8 ± 3.0 minutes, while two cases in the isobaric could reach T4 level within 15 minutes, and the other three could not reach T4 within 30 minutes. So we assumed that the difference of 5 minutes in time to T4 level should be considered clinically significant. At 2-sided type I error of 0.05, 80% power, and pooled SD of 3.76, a sample size of 9.9 for each group was required to detect a 5-minute difference in time to T4 sensory block.

Differences between the groups in quantitative variables were presented as mean and SD and statistically analyzed using Mann-Whitney *U*-test. Categorical data including ASA status, diagnosis, operative procedure, levels of sensory block at 5 and 15 minutes, the peak levels of sensory block, the numbers of patients requiring atropine, vasopressors, or sedatives, and the quality of success were analyzed using Fisher's exact test and presented as number and percentage. The intervals to reach T4 sensory block level were compared using Kaplan-Meier survival curve and log rank test. Using SPSS 17.0 for all statistics, we considered a *P* value <0.05 as statistically significant.

## 3. Results

**Figure 1:**
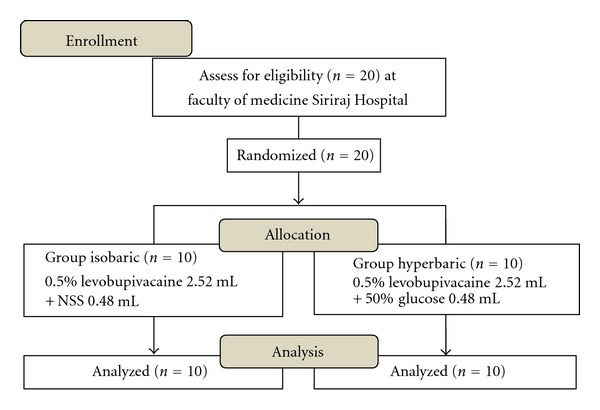
CONSORT flow diagram.

Twenty patients were equally allocated into two groups, the isobaric versus the hyperbaric group (Figure 1). There were no differences with respect to age, height, weight, BMI, ASA status as well as perioperative hemodynamics, amount of IV fluid given, and operation time (Table 1). Diagnoses (myoma, adenomyosis, endometriosis, and ovarian cyst) and types of operation (total abdominal hysterectomy with or without uni/bilateral salpingo-oophorectomy, myomectomy, and ovarian cystectomy) were similar. No serious adverse events happened perioperatively.

**Figure 2:**
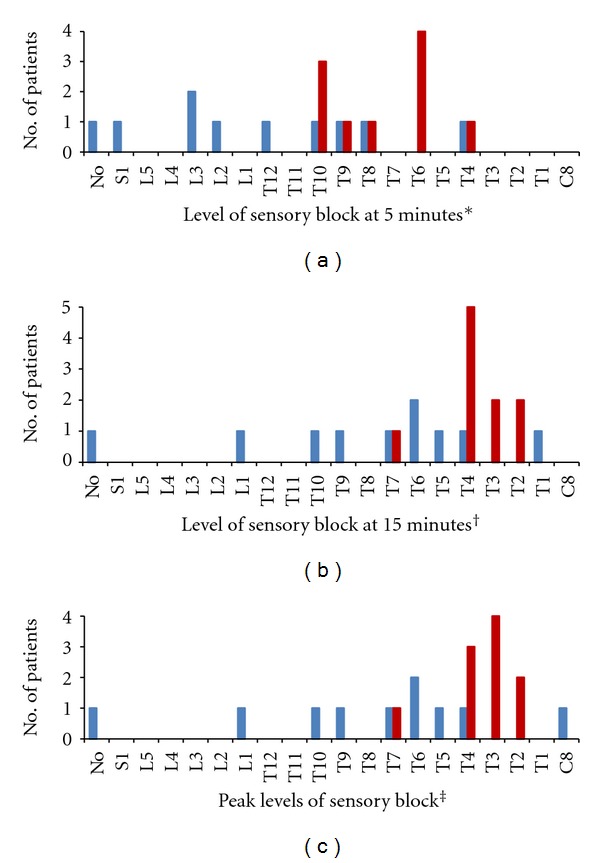
Sensory block levels at 5 and 15 minutes after spinal block, and peak levels of sensory block in patients receiving isobaric (blue bars) or hyperbaric levobupivacaine (red bars). Hyperbaric levobupivacaine reached higher sensory block levels at all periods of time (*P = 0.011, ^†^ *P* = 0.027, and ^‡^ *P* = 0.040, resp.). More patients in hyperbaric group reached T10 or higher at 5 minutes, T4 or higher at 15 minutes, and higher peak levels of sensory block than in isobaric group (*P = 0.011, ^†^ *P* = 0.005, and ^‡^ *P* = 0.005, resp.).

**Figure 3:**
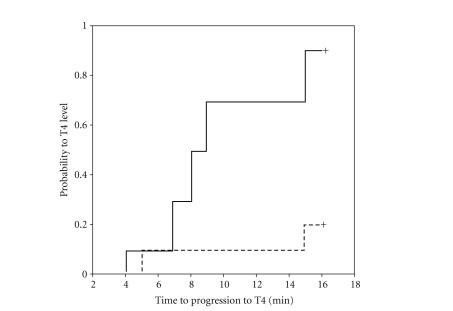
Comparison of time to T4 after isobaric (dotted line) and hyperbaric (dashed line) levobupivacaine (*P* = 0.002).

Hyperbaric levobupivacaine, compared with isobaric levobupivacaine, reached higher sensory block levels at 5 minutes (T8 versus L1, *P* = 0.011) and 15 minutes (T4 versus T7, *P* = 0.027) after injection, and a higher peak sensory block (T4 versus T8, *P* = 0.040). Number of patients in each level of sensory block after hyperbaric compared with isobaric levobupivacaine (Figure 2) are as follows: at five minutes: T10 or higher, ten versus four patients (*P* = 0.011); at 15 minutes: T4 or higher, nine versus two patients (*P* = 0.005); and at the peak sensory block: T4 or higher, nine versus two patients (*P* = 0.005). In three patients sensory spreading continued until 30 minutes (hyperbaric, *n* = 2, T4 to T3 level; and isobaric, T1 to C8 level). One patient in the isobaric group had no sensory block at all, although she had complete motor block (Bromage score 3 at 15 minutes). After Kaplan-Meier survival curve and log rank test, 90% of patients in the hyperbaric group reached T4 sensory block level within 15 minutes while only 20% in the isobaric group did (*P* = 0.002) (Figure 3). As demonstrated in Table 2 significant differences could be identified only in the time intervals reaching T10 level and Bromage score 1. No difference was found for the intervals between spinal injection, and readiness for surgical incision (16.6 versus 17.5 minutes). In patients not converted to alternative anesthesia (nine in the hyperbaric group with T4 and above, and four in the isobaric group with the peak levels at C8, T4, T5, and T6), two-segment regression time, regression time to T10 level, and regression time to modified Bromage score 2 (ability to move feet) were similar. In one hyperbaric group patient, regression time to T10 level could not be determined due to continuous sedation starting 37 minutes after levobupivacaine injection. On arrival at the recovery room (250 minutes after injection), her Bromage score was 0, and she still had no pain.

Twenty percent of patients in the isobaric and 40% in the hyperbaric groups received atropine for bradycardia. Seventy percent of patients in the isobaric and 60% in the hyperbaric groups needed ephedrine for hypotension.

Failure of spinal anesthesia happened in six patients in the isobaric and one in the hyperbaric group (60% versus 10%). Six patients with isobaric levobupivacaine could be admitted to surgery, but two of them needed epidural anesthesia later because the sensory block levels (T7 and T10) did not spread any further and peritoneal stimulation was not tolerated. The other four patients needed epidural anesthesia before starting the operation due to incomplete surgical anesthesia. One patient in the hyperbaric group needed general anesthesia, due to operative difficulties, not to lack of anesthesia. Nine hyperbaric and four isobaric group patients completed the operation with spinal anesthesia, but most of them required additional systemic sedation or analgesia (Table 3).

The quality of surgical anesthesia started to decline at one hour after spinal injection in both groups; only two patients with hyperbaric and one patient with isobaric levobupivacaine assessed the anesthetic quality as “good” (Table 4).

## 4. Discussion

In our study the anesthetic efficiency of 3 mL 0.42% hyperbaric levobupivacaine was superior to 3 mL 0.42% plain levobupivacaine. Patients with hyperbaric solution had a faster onset of sensory and motor block and reached T4 sensory levels, estimated to be sufficient for the planned surgical procedures, faster, and more reliably than with isobaric. Nine patients (90%) in the hyperbaric group underwent surgery completely without additional anesthesia compared with four (40%) in the isobaric group. However, in both groups the anesthetic effect started fading away gradually at one hour after spinal injection. Although surgery could be finished in all of these patients with the help of additional sedatives or analgesics, the outcome was not satisfying.

Sen et al. [5] performed spinal anesthesia with hyperbaric and isobaric levobupivacaine nearly in the same manner as in our study, that is, dosage, concentration, and volume. They could show that hyperbaric levobupivacaine had a faster onset of sensory and motor block, reaching maximum sensory block and Bromage score 3 faster, and had a shorter duration of sensory and motor block than did the isobaric form, except for 2-segment regression time, which were similar in both groups. Contrary to them, we found significant differences only in the onset of sensory and motor block (Table 2). However, the surgery in their study was prostatectomy with transurethral approach, which had less exposure to pain and needed a lower sensory block level (T10) than the level needed for intra-abdominal gynecologic operation (T4) as in our study.

Solakovic [6] investigated isobaric and hyperbaric bupivacaine (15 mg, 0.5%) in patients with orthopedic, urologic, and gynecologic surgery. The hyperbaric agent had a higher peak sensory block level at T5 [T1–T7] compared with T10 [T5–L2] in the isobaric group but led to a high block with consecutive hemodynamic instability in some patients. Xu et al. [7] reported patients undergoing lower abdominal surgery who received spinal injection of either hyperbaric or isobaric bupivacaine (15 mg, 0.5%) in lateral decubitus position and then were shifted to supine position similar to our study. They found a higher sensory peak level and shorter time to achieve it, a shorter sensory and motor block regression time, a longer recovery time for urination function, and higher incidences of side effects, with the hyperbaric solution, and concluded that isobaric bupivacaine was superior to its hyperbaric form. Camponovo et al. [8] compared 40 mg and 60 mg 2% hyperbaric with 60 mg 2% plain prilocaine for spinal anesthesia in outpatient surgery. The hyperbaric solutions had faster onset times, higher peak level, shorter duration of surgical block, and faster time to urination than did the plain solution, suggesting its superiority for the ambulatory setting. Luck et al. [9] compared 3 mL of 0.5% hyperbaric levobupivacaine, bupivacaine, and ropivacaine for spinal anesthesia. Levobupivacaine and bupivacaine were clinically the same while ropivacaine had shorter duration of sensory and motor block.

The differences in our study may partly be due to different baricity of the solutions used. Baricity is a measure of the relative density of local anesthetic solution when compared with CSF. According to Hocking and Wildsmith [10] local anesthetics which have baricity which ranges from 0.9990 to 1.0010 are isobaric; so 0.42% plain levobupivacaine used in our study is considered isobaric, as both baricities of 0.25% and 0.5% plain levobupivacaine are within these isobaric ranges (calculated from the density of plain levobupivacaine at 37°C reported by McLeod [11] divided by the density of CSF at 37°C, reported by Lui et al. [12]). Since opioids, except pethidine, are hypobaric [12], so we avoided injecting morphine together with levobupivacaine intrathecally but provided epidurally later for postoperative analgesia instead.

Gori et al. [13] suggested that isobaric levobupivacaine in CSF acts indifferently to gravitational forces, both immediately after the injection and later on. Therefore levels of sensory block after intrathecal isobaric levobupivacaine are unaffected by the patient position following the injection. This might be an advantage over plain bupivacaine which has the tendency to spread unexpectedly high even after a reasonable time for fixation [14, 15] thus causing late complications such as hypotension and bradycardia from high block. Ariyama et al. [16] reported differently to Gori's that the duration in the sitting position after injection, not the baricity, affected the cephalad spread of 0.5% bupivacaine, but they used only small volumes (1 mL) of 0.5% iso- and hyperbaric bupivacaine. In our study the highest sensory level (C8) occurred in one patient with isobaric levobupivacaine. As we did control the position of the patients during and after injection, the dose of the anesthetic injected, as well as other factors influencing the spread, that is, patients characteristics and technique of injection, the variation of cephalad spread could be due to baricity [2, 6, 15–18].

The minimum local anesthetic dose (MLAD) of levobupivacaine is 10.58 mg for Cesarean section [19] and 5.68 mg for lower limb surgery [20]. As there are no data available, we estimated that 12.6 mg would be appropriate for patients with lower abdominal incision. Our data suggest that gynecological surgery with lower abdominal approach and a mean duration of about 2 hours needs sensory blockade at T4 level. Nine patients (90%) in the hyperbaric group could achieve T4 level, compared with two (20%) in the isobaric group. Nine patients (90%) in the hyperbaric group were able to start and finish surgery with spinal anesthesia, with some analgesics and sedatives supplement, compared with four patients (40%) in the isobaric group. As quality of surgical anesthesia declined after an hour independently of the agent used, we concluded that the dosage given was too low to provide analgesia up to T4 sensory level long time enough. Further studies should be done to find the optimal dose and to find whether a higher site of needle insertion or a faster rate of injection with measures correlated to gravity, for example, a head-up or head-down position, can provide better anesthesia for intra-abdominal surgery, which requires a sensory block level up to T4 and may last up to three hours. Continuous spinal anesthesia suggested by Möllmann [21] may be an interesting alternative, achieving both adequate anesthesia and improved control of cephalad spread.

The data of our study suggest better anesthesia with hyperbaric levobupivacaine compared with its isobaric form in intra-abdominal surgery that lasts not more than one hour. The wide variation of peak sensory block levels of plain levobupivacaine (L1 to C8) makes its effect markedly less predictable than that of the hyperbaric form (T7 to T2). Nevertheless, levobupivacaine could be an enrichment within the anesthetic arena, at least in its hyperbaric form.

## Figures and Tables

**Table 1:** Characteristics of patients receiving spinal anesthesia with isobaric or hyperbaric levobupivacaine for gynecologic surgery. Values are mean ± SD [min–max] or number of patients.

	Isobaric (*n* = 10)	Hyperbaric (*n* = 10)	*P* value
Age (yr)	44.8 ± 6.3 [34–55]	42.6 ± 7.3 [28–50]	0.592
Body weight (kg)	59.4 ± 8.5 [48–74]	54.9 ± 9.1 [40–69]	0.342
Height (cm)	158.1 ± 3.2 [154–165]	157.5 ± 5.6 [150–171]	0.484
BMI (kg/m^2^)	23.8 ± 3.7 [17.6–30.4]	22.1 ± 3.3 [16.6–27.5]	0.342
ASA class I : II (n)	8 : 2	8 : 2	
Average baseline systolic BP (mmHg)	129 ± 7 [120–140]	121 ± 10 [105–134]	0.143
Average baseline diastolic BP (mmHg)	76 ± 9 [63–89]	70 ± 7 [60–79]	0.172
Average baseline heart rate (bpm)	84 ± 13 [63–106]	84 ± 17 [68–117]	0.796
Amount of IV fluid in 15 minutes before levobupivacaine injection (mL)	690 ± 139 [500–850]	651 ± 126 [500–810]	0.587
Operation time (min)	94 ± 26 [51–135]	110 ± 49 [45–213]	0.684

**Table 2:** Characteristics of intrathecal blocks with isobaric or hyperbaric levobupivacaine in patients undergoing gynecologic surgery. Values are number of patients in each group, or minutes in mean ± SD [min–max].

	Isobaric: Hyperbaric (*n*)	Isobaric (min)	Hyperbaric (min)	*P* value
Time to T10 sensory block	8 : 10	6.6 ± 4.7 [2–15]	2.8 ± 1.1 [1–5]	**0.039**
Time to T4 sensory block	2 : 9	10.0 ± 7.1 [5–15]	9.1 ± 3.7 [4–15]	
Time to peak sensory block	9 : 10	13.8 ± 6.8 [8–30]	15.9 ± 8.1 [7–30]	0.517
Time to modified Bromage score 1	10 : 10	6.9 ± 5.3 [2–20]	2.9 ± 2.9 [1–10]	**0.013**
Time to modified Bromage score 3	9 : 10	13.6 ± 7.3 [5–30]	8.2 ± 6.8 [1–24]	0.064
Time to incision	6 : 10	17.5 ± 4.6 [11–25]	16.6 ± 4.9 [12–27]	0.623
2-segment regression time	4 : 9	98.3 ± 29.5 [60–123]	110.8 ± 42.9 [60–192]	
Regression time to T10	4 : 8	160.0 ± 50.4 [100–222]	158.9 ± 60.0 [91–262]	
Regression time to modified Bromage score 2	4 : 9	143.3 ± 74.7 [60–240]	102.4 ± 20.9 [70–131]	

Values are compared with Mann-Whitney *U*-test.

**Table 3:** Reasons for supplemental drugs and convenience in performing gynecological surgery with abdominal incision in patients receiving intrathecal blocks with isobaric or hyperbaric levobupivacaine. Values are number (proportion).

	Isobaric (*n* = 10)	Hyperbaric (*n* = 10)
Medication for		
(i) Anxiety	1 (10)	5 (50)
(ii) Inadequate analgesia	9 (90)	3 (30)
(iii) Surgical difficulty		1 (10)
(iv) No need		1 (10)
Convenience for surgeons		
(i) Impossibility to start operation	4 (40)	0
(ii) Impossibility to continue operation	2 (20)	1 (10)
(iii) Some difficulty during operation	3 (30)	2 (20)
(iv) Satisfactory	1 (10)	7 (70)

**Table 4:** Quality of surgical anesthesia graded by patients at incision time, at 1 hour and 2 hours after injection of local anesthetic, and at skin suture time. The intervals between skin incision and suture time in isobaric and hyperbaric groups were [min–max] [51–135] minutes and [45–213] minutes, respectively. Values are number of patients.

			Quality of surgical anesthesia				
Time	Group	*n*	Worst	Poor	Fair	Good	Sedated
Skin incision	I*	7	1	0	2	4	0
	H^†^	10	0	0	3	7	0
1 hour after injection	I*	4	0	0	2	1	1
	H^†^	9	0	2	4	2	1
2 hours after injection	I*	2	0	1	1	0	0
	H^†^	5	0	1	3	0	1
Skin suture	I*	4	0	1	2	0	1
(time varied)	H^†^	9	0	2	5	1	1

Worst: discomfort because of pain; Poor: discomfort, feeling intense pressure or traction; Fair: comfortable but experiencing pressure and traction; Good: comfortable without any feeling; Sedated: no grading possible. I*, Isobaric; H^†^, Hyperbaric.

## References

[1] Camorcia M, Capogna G, Berritta C, Columb MO (2007). The relative potencies for motor block after intrathecal ropivacaine, levobupivacaine, and bupivacaine. *Anesthesia and Analgesia*.

[2] Cappelleri G, Aldegheri G, Danelli G (2005). Spinal anesthesia with hyperbaric levobupivacaine and ropivacaine for outpatient knee arthroscopy: a prospective, randomized, double-blind study. *Anesthesia and Analgesia*.

[3] Casati A, Moizo E, Marchetti C, Vinciguerra F (2004). A prospective, randomized, double-blind comparison of unilateral spinal anesthesia with hyperbaric bupivacaine, ropivacaine, or levobupivacaine for inguinal herniorrhaphy. *Anesthesia and Analgesia*.

[4] Vanna O, Chumsang L, Thongmee S (2006). Levobupivacaine and bupivacaine in spinal anesthesia for transurethral endoscopic surgery. *Journal of the Medical Association of Thailand*.

[5] Sen H, Purtuloglu T, Sizlan A (2010). Comparison of intrathecal hyperbaric and isobaric levobupivacaine in urological surgery. *Minerva Anestesiologica*.

[6] Solakovic N (2010). Level of sensory block and baricity of bupivacaine 0.5% in spinal anesthesia. *Medicinski Arhiv*.

[7] Xu L, Guo QL, Yan JQ (2005). Isobaric and hyperbaric local anesthetic used in spinal anesthesia. *Zhong Nan Da Xue Xue Bao Yi Xue Ban*.

[8] Camponovo C, Fanelli A, Ghisi D, Cristina D, Fanelli G (2010). A prospective, double-blinded, randomized, clinical trial comparing the efficacy of 40 Mg and 60 Mg hyperbaric 2% prilocaine versus 60 Mg plain 2% prilocaine for intrathecal anesthesia in ambulatory surgery. *Anesthesia and Analgesia*.

[9] Luck JF, Fettes PDW, Wildsmith JAW (2008). Spinal anaesthesia for elective surgery: a comparison of hyperbaric solutions of racemic bupivacaine, levobupivacaine, and ropivacaine. *British Journal of Anaesthesia*.

[10] Hocking G, Wildsmith JAW (2004). Intrathecal drug spread. *British Journal of Anaesthesia*.

[11] McLeod GA (2004). Density of spinal anaesthetic solutions of bupivacaine, levobupivacaine, and ropivacaine with and without dextrose. *British Journal of Anaesthesia*.

[12] Lui ACP, Polis TZ, Cicutti NJ (1998). Densities of cerebrospinal fluid and spinal anaesthetic solutions in surgical patients at body temperature. *Canadian Journal of Anaesthesia*.

[13] Gori F, Corradetti F, Cerotto V, Peduto VA (2010). Influence of positioning on plain levobupivacaine spinal anesthesia in cesarean section. *Anesthesiology Research and Practice*.

[14] Niemi L, Tuominen M, Pitkänen M, Rosenberg PH (1993). Effect of late posture change on the level of spinal anaesthesia with plain bupivacaine. *British Journal of Anaesthesia*.

[15] Vicent O, Litz RJ, Hübler M, Koch T (2003). Secondary cranial extension after spinal anesthesia with isobaric 0.5% bupivacaine following postural change. *Anaesthesist*.

[16] Ariyama J, Hayashida M, Sugimoto Y, Imanishi H, To-Oyma Y, Kitamura A (2009). Spread of spinal anesthesia in patients having perianal surgery in the jackknife position: effects of baricity of 0.5% bupivacaine and positioning during and after induction of spinal anesthesia. *Journal of Clinical Anesthesia*.

[17] Danelli G, Baciarello M, Di Cianni S (2008). Effects of baricity of 0.5% or 0.75% levobupivacaine on the onset time of spinal anesthesia: a randomized trial. *Canadian Journal of Anesthesia*.

[18] Wildsmith JAW (2010). Density matters most. *Anaesthesia*.

[19] Parpaglioni R, Frigo MG, Lemma A, Sebastiani M, Barbati G, Celleno D (2006). Minimum local anaesthetic dose (MLAD) of intrathecal levobupivacaine and ropivacaine for Caesarean section. *Anaesthesia*.

[20] Lee YY, Ngan Kee WD, Fong SY, Liu JT, Gin T (2009). The median effective dose of bupivacaine, levobupivacaine, and ropivacaine after intrathecal injection in lower limb surgery. *Anesthesia and Analgesia*.

[21] Möllmann M (1997). Continuous spinal anesthesia. *Anaesthesist*.

